# Association between Modic changes and recurrence of lumbar disc herniation after percutaneous endoscopic lumbar discectomy: a meta-analysis

**DOI:** 10.3389/fsurg.2025.1694557

**Published:** 2025-11-27

**Authors:** Xianfeng Li, Honghong Ren, Lipeng Peng, Jiaqin Yuan

**Affiliations:** 1 Department of Orthopedics, The Second People’s Hospital of Yibin, Yibin, Sichuan, China; 2 Department of Nephrology, The Second People’s Hospital of Yibin, Yibin, Sichuan, China

**Keywords:** Modic changes, lumbar disc herniation, percutaneous endoscopic lumbar discectomy, recurrence, meta-analysis

## Abstract

**Purpose:**

This study aims to identify the association between preoperative Modic changes and the recurrence of lumbar disc herniation (LDH) in patients who have undergone percutaneous endoscopic lumbar discectomy (PELD).

**Methods:**

The PubMed, Web of Science, EMBASE, and CNKI databases were searched from their inception until 19 March 2025. Early recurrence was defined as herniation occurring within 6 months postoperatively, whereas late recurrence referred to herniation occurring after 12 months. Odds ratios (ORs) with 95% confidence intervals (CIs) were combined, and subgroup analyses were conducted according to the recurrence type.

**Results:**

Twenty-seven studies involving 10,116 patients were included, with the majority of studies originating from China (25/27). The recurrence rates for patients without and with Modic changes were 7.44% and 16.41%, respectively (type I: 15.01%; type II/III: 18.14%; *P* < 0.001). The presence of Modic changes was associated with a significantly increased risk of recurrence (OR = 2.96, 95% CI: 2.29–3.82, *P* < 0.001), and subgroup analyses by the recurrence period (early or late) showed consistent findings. However, patients with Modic type II/III changes did not have a higher risk of recurrence than those with Modic type I changes (OR = 1.13, *P* = 0.217).

**Conclusion:**

Preoperative Modic changes are associated with postoperative recurrence among LDH patients undergoing PELD, and the presence of Modic changes is related to a significantly higher risk of early and late recurrence.

## Introduction

Lumbar disc herniation (LDH) is one of the most common degenerative spinal disorders, primarily caused by degeneration or external stress that leads to the nucleus pulposus protruding through the annulus fibrosus and compressing adjacent nerve roots (1). This condition often results in low back pain, radiculopathy, and, in some cases, motor or sensory dysfunction (2). The incidence of LDH has been increasing steadily and is considered a major contributor to reduced quality of life and work capacity among the working-age population. Epidemiological studies have shown that approximately 60%–80% of adults experience low back pain at some point in their lives, with LDH being one of the leading causes (3). Although conservative treatments—such as pharmacotherapy, physical therapy, and spinal traction—may offer symptom relief in the early stages, surgical intervention remains the most effective treatment option for patients with persistent or worsening symptoms unresponsive to conservative management (4). Common surgical techniques include open discectomy, microdiscectomy, and minimally invasive procedures such as percutaneous endoscopic lumbar discectomy (PELD). These procedures generally provide rapid symptom relief and significantly improve patients' quality of life. However, postoperative recurrence remains a clinical concern, with reported recurrence rates ranging from 5% to 15% (5). Recurrence is often associated with inadequate rehabilitation, residual disc fragments, or further degeneration of the intervertebral disc. Therefore, while surgical treatment offers favorable short-term outcomes, postoperative rehabilitation and long-term management are equally important to prevent recurrence and ensure sustained recovery.

Recent studies have suggested a potential association between Modic changes—vertebral endplate and bone marrow signal alterations detected on magnetic resonance imaging (MRI)—and postoperative recurrence following PELD (6). Modic changes, commonly classified into three types (type I: inflammatory, type II: fatty degeneration, and type III: sclerosis), are considered imaging indicators of degenerative changes at the vertebral endplate–disc interface (6). These changes are increasingly observed in patients with LDH and are thought to reflect underlying pathological processes such as endplate damage, inflammatory responses, and biomechanical alterations (7).

Although some studies have indicated that the presence of Modic changes may be associated with an increased risk of recurrent disc herniation after PELD, the existing evidence remains inconclusive and somewhat inconsistent. Therefore, we conducted a meta-analysis to further clarify the relationship between preoperative Modic changes and postoperative recurrence in patients undergoing PELD, aiming to provide a more robust evidence base for clinical decision-making and surgical risk assessment.

## Materials and methods

This meta-analysis was conducted in accordance with the 2020 Preferred Reporting Items for Systematic Reviews and Meta-Analyses (8).

### Literature search

The PubMed, EMBASE, CNKI, and Web of Science databases were searched from their inception until 19 March 2025. The following terms were used in the search: lumbar disc herniation, LDH, percutaneous endoscopic lumbar discectomy, percutaneous transforaminal endoscopic discectomy, PETD, PED, recurrence, and Modic. A detailed search PubMed strategy is shown in Supplementary File S1. Meanwhile, MeSH terms and free texts were applied. References for the included studies were also reviewed.

### Inclusion and exclusion criteria

Studies were included if they met the following criteria: (1) patients were diagnosed with LDH and underwent PELD; (2) the presence or absence of Modic changes was evaluated before the surgery by MRI according to previously reported criteria (9); (3) recurrence rates for patients with and without Modic (type I, II, or III) changes were reported; (4) detailed information was provided for the calculation of odds ratios (ORs) with 95% confidence intervals (CIs) to investigate the association between Modic changes and recurrence risk; (5) the study was published in English or Chinese; (6) the full text was available.

Studies were excluded if they met any of the following criteria: (1) contained overlapping or duplicate data; or (2) were meeting abstracts, letters, animal trials, editorials, reviews, or case reports.

### Data extraction

We extracted the following data from each included study: first author, publication year, country, sample size, follow-up duration, number of patients, and number of patients experiencing recurrence with non-Modic changes, type I Modic change, type II Modic change, and type III Modic change, and ORs and 95% CIs.

In this meta-analysis, early recurrence was defined as recurrence occurring within 6 months after surgery (10), and late recurrence was defined as recurrence occurring after 12 months (11).

### Quality assessment

All included studies were cohort studies, and the Newcastle–Ottawa Scale (NOS) was used to assess their methodological quality (12). Studies with an NOS score ≥6 were defined as high-quality.

The literature search, study selection, data collection, and quality assessment were independently conducted by two authors (XL and HR), and any disagreements were resolved through consensus or consultation with a third reviewer (LP).

### Statistical analysis

In our study, all statistical analyses were performed using STATA 17.0 software. Heterogeneity among the included studies was evaluated using the *I*^2^ statistic and the *Q*-test. When significant heterogeneity was detected (*I*^2^ > 50% and/or *P* < 0.1), a random-effects model was applied; otherwise, a fixed-effects model was used. ORs and 95% CIs were calculated to evaluate the association between Modic changes and recurrence risk. Subgroup analyses based on the recurrence period were also performed. Sensitivity analyses were performed to identify potential sources of heterogeneity and assess the stability of the pooled results. In addition, Begg's funnel plot and Egger's test were conducted to detect publication bias, with significant publication bias defined as *P* < 0.05 (13, 14). If significant publication bias was detected, the trim-and-fill method was applied to identify potentially unpublished studies (15).

## Results

### Literature search and selection process

**Figure 1:**
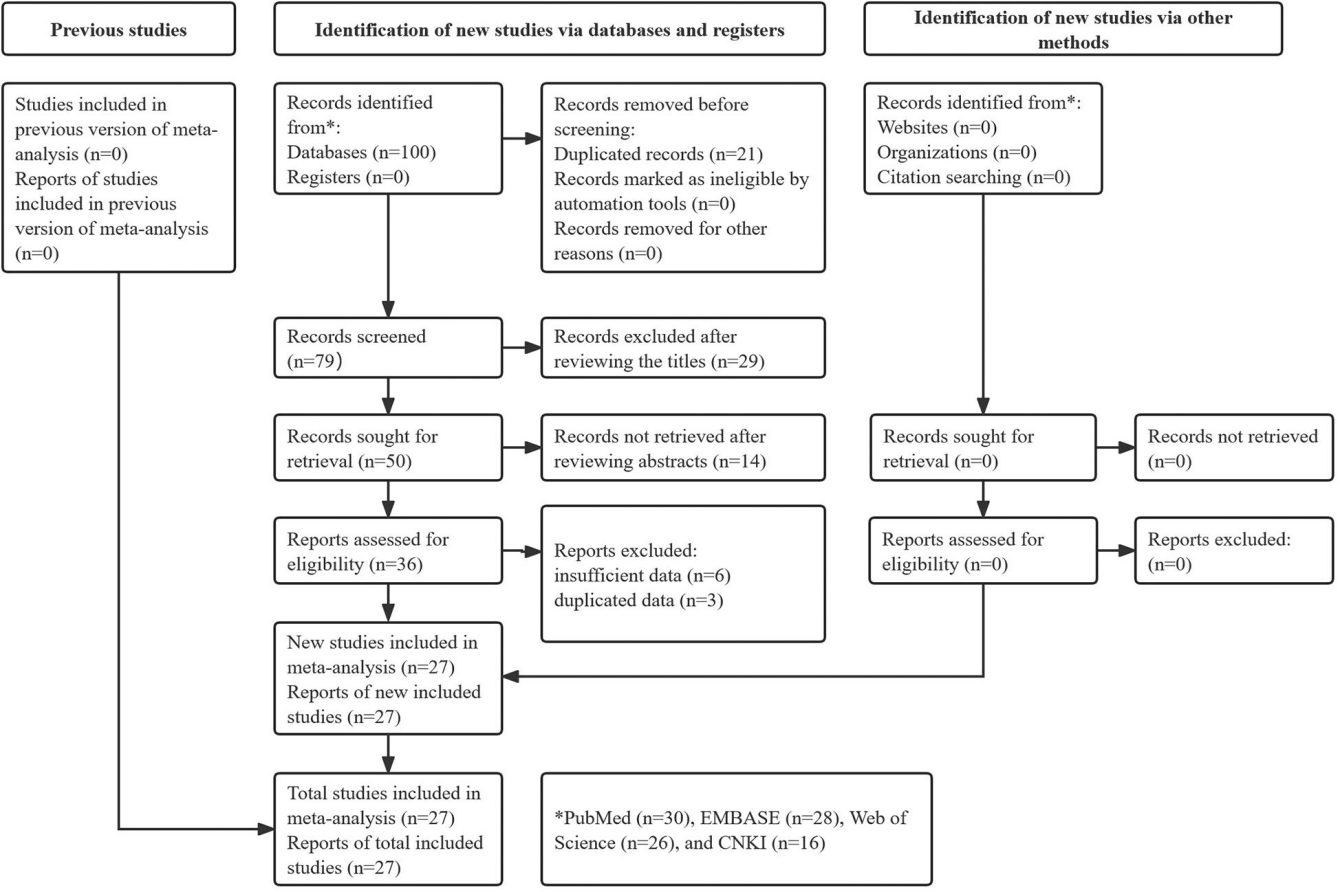
The PRISMA flow diagram of this study.

A total of 100 records were identified through searches of the four databases, and 21 duplicate records were removed. After reviewing the titles, abstracts, and full texts, 29, 14, and 9 publications were excluded, respectively. Eventually, 27 studies were included in this meta-analysis (10, 16–41) (Figure 1).

### Basic characteristics of included studies

Among the 27 included studies, 10,116 patients were enrolled, with sample sizes ranging from 84 to 1,807. Most studies were conducted in China (25/27). Six studies focused on early recurrence (within 6 months), while 15 examined late recurrence (after 12 months). All of the included studies were deemed high quality. Specific data are presented in Table 1.

**Table 1:** Basic characteristics of included studies.

Author	Year	Country	Sample size	Follow-up time (months)	Non-Modic changes (*n*)		Type I Modic change (*n*)		Type II Modic change (*n*)		Type III Modic change (*n*)		NOS
					Recurrence	Overall	Recurrence	Overall	Recurrence	Overall	Recurrence	Overall	
Kim (16)	2007	Korea	84	0.5–32	NR	NR	NR	NR	NR	NR	NR	NR	6
Yao (17)	2017	China	116	1–72	NR	NR	NR	NR	NR	NR	NR	NR	6
Liu (18)	2018	China	212	12–36	2	108	9	104	0	0	0	0	7
Chen (19)	2019	China	218	24–40	3	133	0	2	9	71	0	4	7
Kim (20)	2019	Republic of Korea	300	6	23	278	3	12	1	7	1	3	7
Xu (21)	2019	China	276	≥24 months	8	182	4	44	4	50	0	0	7
Hao (22)	2020	China	99	24–60	2	71	2	9	4	19	0	0	6
Jia (10)	2021	China	352	6	16	265	2	6	13	78	1	3	8
Li (23)	2021	China	1,807	≥60	63	1,271	5	56	109	366	0	13	8
Liu (24)	2021	China	209	24	3	129	9 (recurrence)			80 (overall)			7
Zhao (25)	2021	China	286	≥12	36	181	5	21	21	83	1	1	8
Guan (26)	2022	China	357	25.33 ± 9.48	24	293	5 (recurrence)			64 (overall)			7
Qin (27)	2022	China	142	≥12	2	62	5	32	7	48	0	0	6
Shu (28)	2022	China	65	12	2	52	5 (recurrence)			13 (overall)			6
Zhang (29)	2022	China	197	6	2	137	8 (recurrence)			60 (overall)			6
Zhu (30)	2022	China	249	12–48	1	115	0	0	9	134	0	0	7
He (31)	2023	China	690	24	6	251	20	137	35	284	2	18	7
Li (32)	2023	China	645	24.21 ± 8.22	12	378	11	71	33	196	0	0	7
Shi (33)	2023	China	207	12	3	117	2	23	4	67	0	0	7
Tan (34)	2023	China	718	≥9	14	395	3	62	17	239	2	22	8
Tang (35)	2023	China	228	6	7	138	2	11	14	77	0	1	7
Li (36)	2024	China	285	6	9	239	2	7	7	36	1	3	7
Luo (37)	2024	China	132	6	7	114	2	4	3	14	0	0	6
Pan (38)	2024	China	300	≥12	101	237	17	26	27	32	5	5	7
Ren (39)	2024	China	1,159	NR	48	567	82 (recurrence)			592 (overall)			8
Shan (40)	2024	China	171	NR	42	147	1	6	12	15	1	2	6
Tang (41)	2024	China	612	7.99 ± 1.29	NR	NR	NR	NR	NR	NR	NR	NR	7

NR, not reported; NOS, Newcastle–Ottawa Scale.

### Recurrence rates in LDH patients after PELD

First, we calculated the recurrence rates of LDH in patients with and without Modic changes. The results showed that the recurrence rate in patients with Modic changes (16.41%, 547/3,333) was significantly higher than that in patients without Modic changes (7.44%, 436/5,424) (*P* < 0.001). In detail, the recurrence rates in patients with type I and type II/III Modic changes were 15.01% (95/633) and 18.14% (343/1,891), respectively (*P* = 0.072).

### Association between preoperative Modic changes and recurrence in LDH patients receiving PELD

**Figure 2:**
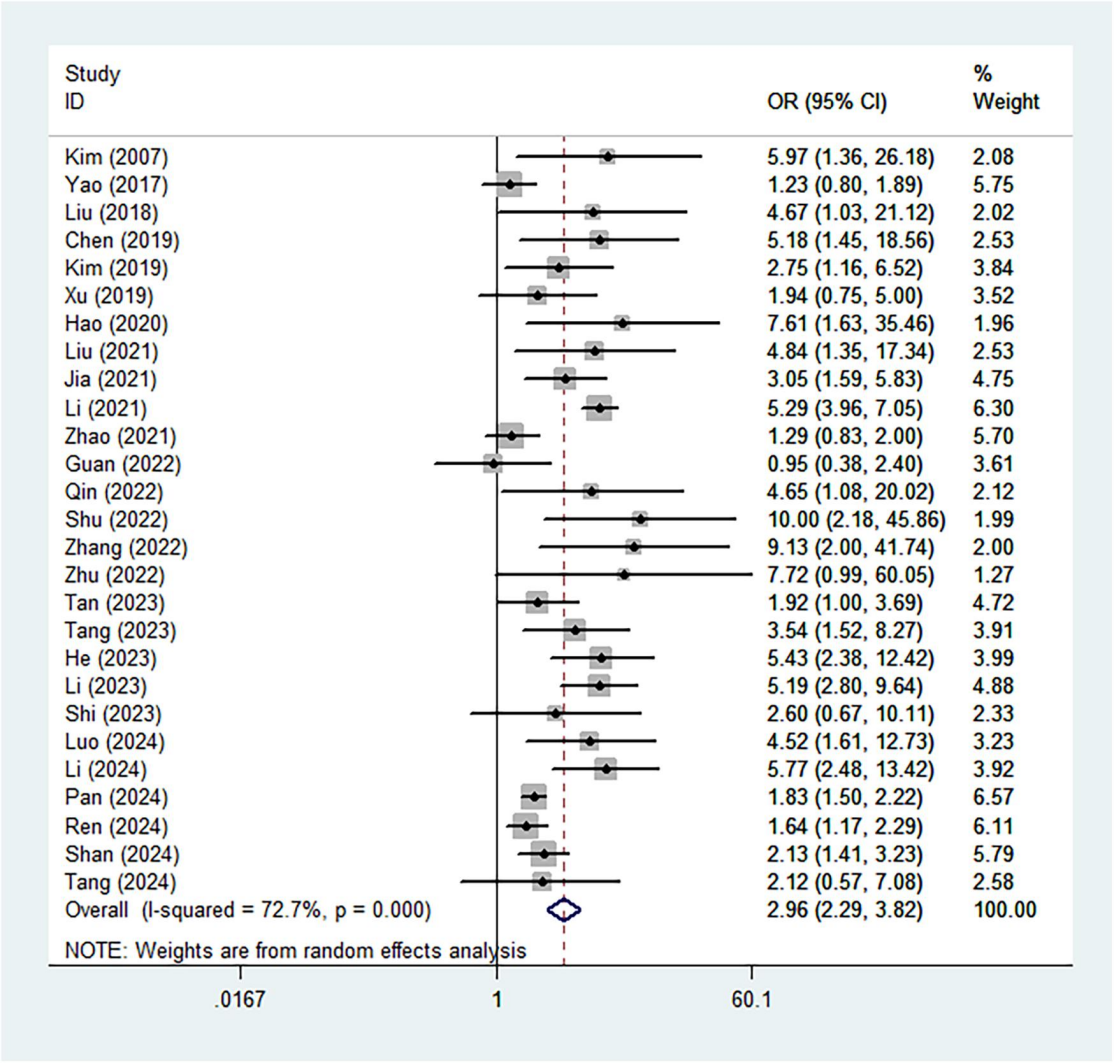
Association between the presence of Modic changes and postoperative recurrence in patients with lumbar disc herniation after the percutaneous endoscopic lumbar discectomy.

Based on the pooled results of the meta-analysis, the presence of Modic changes was significantly associated with an increased risk of recurrence (OR = 2.96, 95% CI: 2.29–3.82, *P* < 0.001; *I*^2^ = 72.7%, *P* < 0.001) (Figure 2). Subgroup analysis by the recurrence period showed similar findings (late: OR = 3.36, 95% CI: 2.23–5.04, *P* < 0.001; early: OR = 3.84, 95% CI: 2.69–5.48, *P* < 0.001) (Supplementary Figure S1).

**Figure 3:**
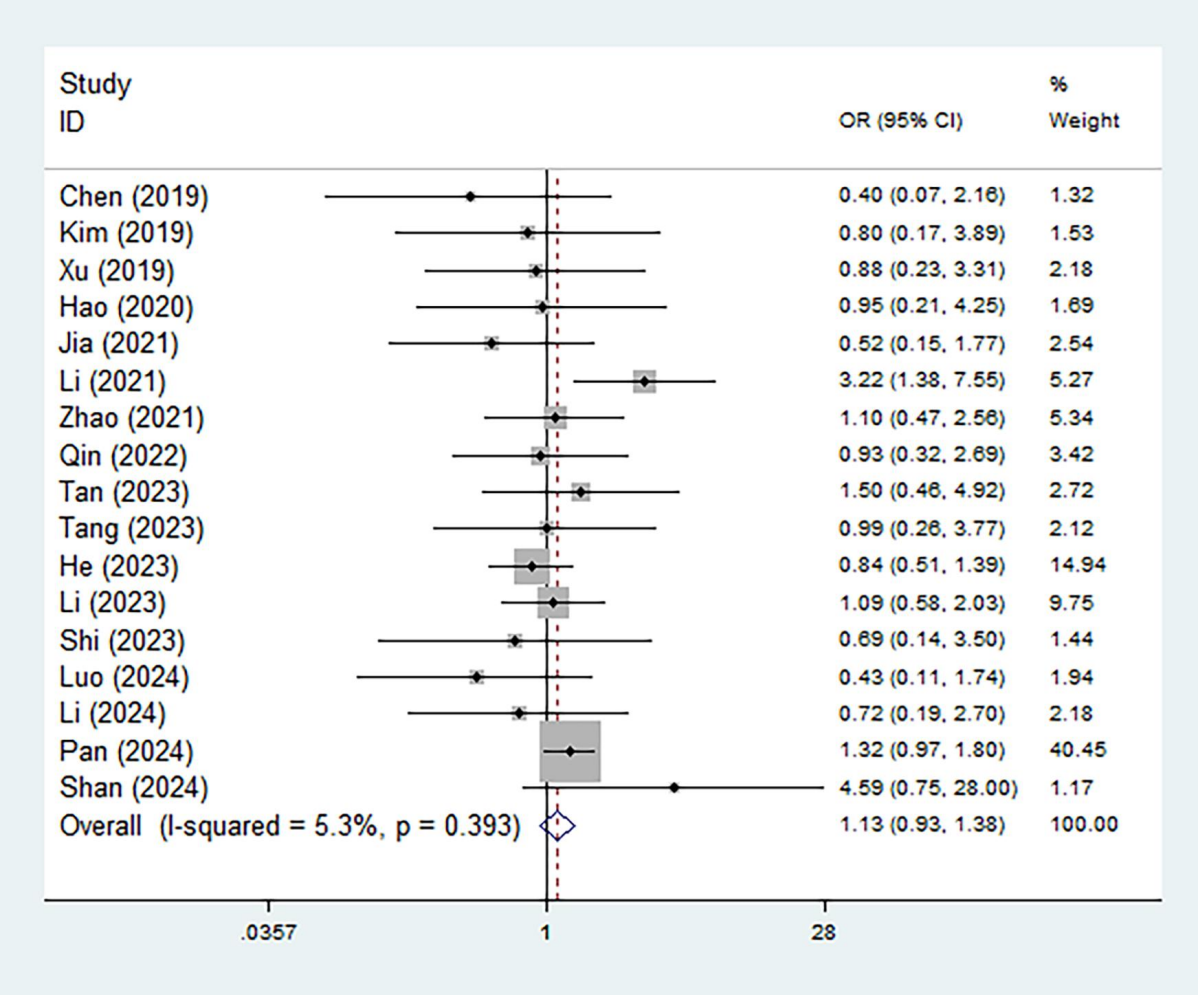
Association between the type of Modic changes (II/III vs. I) and postoperative recurrence in patients with lumbar disc herniation after the percutaneous endoscopic lumbar discectomy.

In addition, the association between different types of Modic changes and recurrence risk was also explored. However, the recurrence rates of LDH in patients with type I versus type II/III Modic changes were not statistically different (OR = 1.13, 95% CI: 0.93–1.38, *P* = 0.217) (Figure 3). Subgroup analyses based on the recurrence period yielded consistent results (late: OR = 1.17, 95% CI: 0.95–1.45, *P* = 0.135; early: OR = 0.65, 95% CI: 0.36–1.20, *P* = 0.170) (Supplementary Figure S2).

### Sensitivity analysis

**Figure 4:**
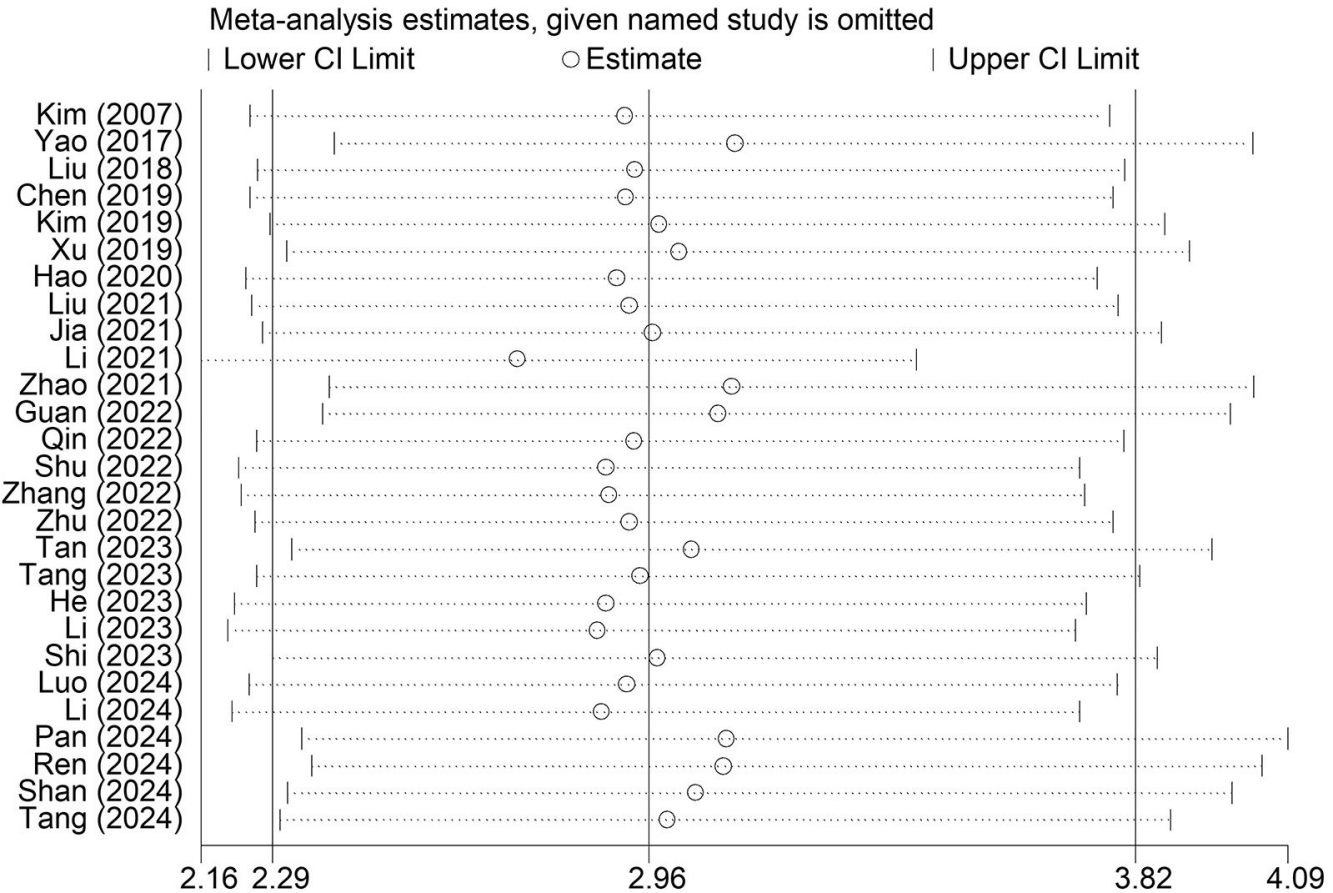
Sensitivity analysis for the association between the presence of Modic changes and postoperative recurrence in patients with lumbar disc herniation after the percutaneous endoscopic lumbar discectomy.

Sensitivity analysis indicated that the results were stable and reliable, and no individual study had a significant impact on the overall findings (Figure 4).

### Publication bias

**Figure 5:**
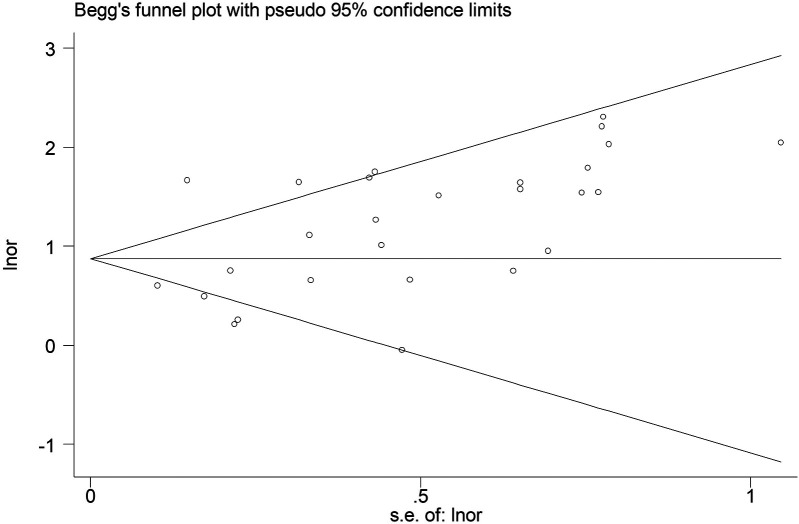
Begg's funnel plot for the association between the presence of Modic changes and postoperative recurrence in patients with lumbar disc herniation after the percutaneous endoscopic lumbar discectomy.

**Figure 6:**
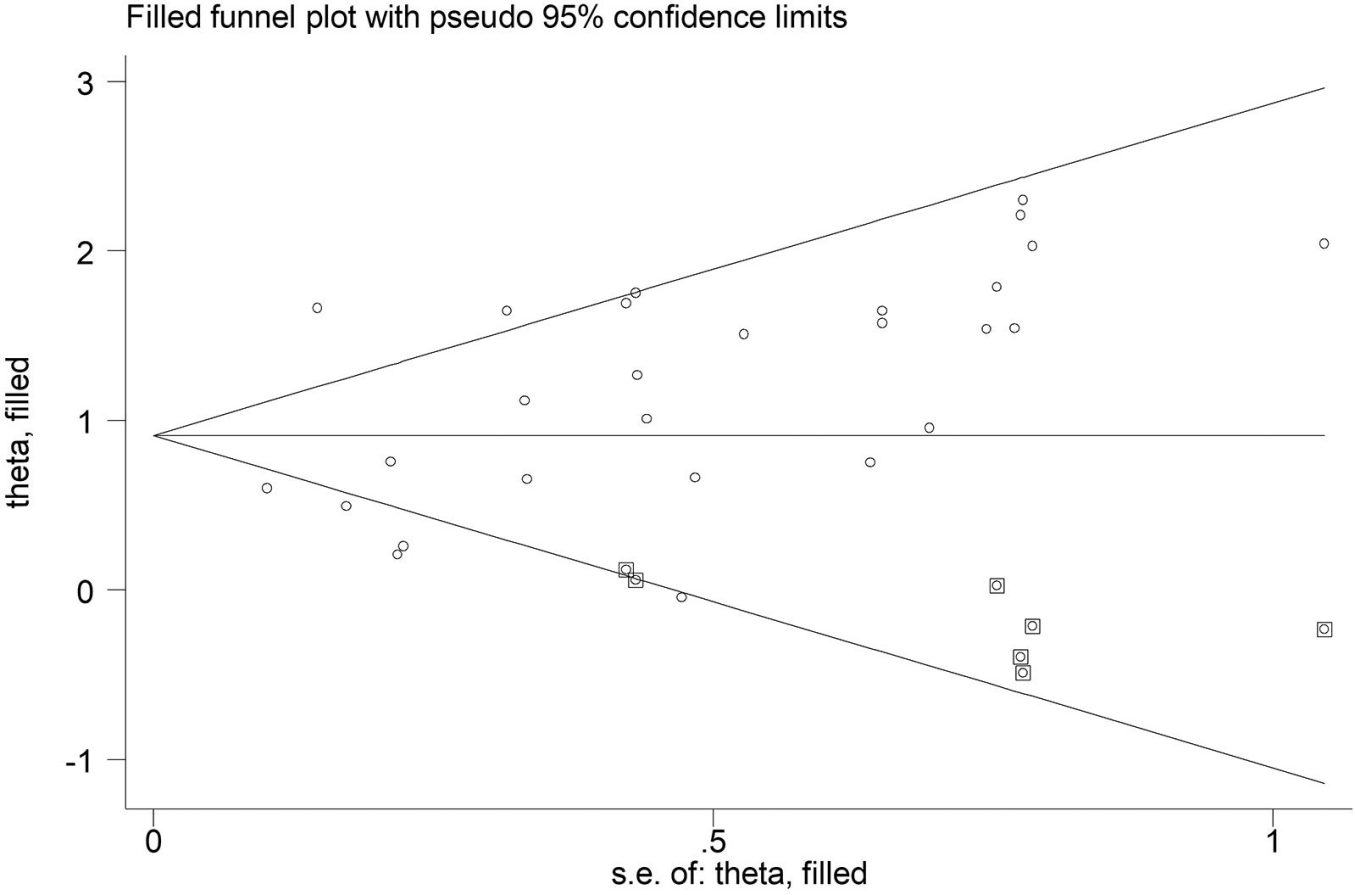
Filled Begg's funnel plot for the association between the presence of Modic changes and postoperative recurrence in patients with lumbar disc herniation after the percutaneous endoscopic lumbar discectomy.

According to Begg's funnel plot (Figure 5) and Egger's test (*P* = 0.039), obvious publication bias was detected. Therefore, the trim-and-fill method was applied, revealing seven potentially unpublished studies (Figure 6). However, these seven studies did not affect the overall conclusion (random-effects filled OR = 2.49, 95% CI: 1.96–3.16, *P* < 0.001; fixed-effects filled OR = 2.28, 95% CI: 2.05–2.54, *P* < 0.001).

## Discussion

In the current meta-analysis, we included 27 studies with 10,116 patients and evaluated the association between preoperative Modic changes and postoperative recurrence of LDH among patients undergoing PELD. Our pooled results indicated that the presence of Modic changes, regardless of the type, was significantly associated with an increased risk of postoperative recurrence. Therefore, preoperative evaluation of Modic changes is essential for LDH patients. However, due to the limitations of the included studies, more prospective cohort studies or randomized trials are needed to verify our findings.

Several mechanisms have been proposed to explain why patients with LDH accompanied by Modic changes may exhibit a higher risk of postoperative recurrence following PELD. First, Modic changes often reflect structural damage to the vertebral endplates, which can lead to intervertebral segmental instability. This biomechanical alteration may expose the operated disc to increased mechanical stress, thereby accelerating disc degeneration and increasing the likelihood of reherniation (42). Second, particularly in patients with Modic type I changes, a persistent inflammatory microenvironment around the endplate and bone marrow may not resolve following surgical decompression. Such inflammation may contribute to ongoing degeneration and residual or recurrent symptoms (43). In addition, Modic changes are commonly associated with advanced disc degeneration, including reduced water content, annular fissures, and fragmentation of the nucleus pulposus. These degenerative changes can impair the disc's ability to structurally recover after surgery, making it more susceptible to recurrent herniation (44). Moreover, in patients with Modic changes, the annulus fibrosus is often more severely compromised, which may lead to incomplete repair of annular defects and residual disc fragments postoperatively—factors that have been linked to recurrence (45). Finally, Modic-related alterations in load transmission across the vertebral body may cause abnormal stress redistribution, further promoting recurrent disc protrusion either at the surgical level or at adjacent segments (46). Although these mechanisms are not fully elucidated, they highlight the potential role of Modic changes in influencing surgical outcomes and underscore the need for careful preoperative assessment and postoperative management in this patient population.

Beyond their potential association with postoperative recurrence, preoperative assessment of Modic changes may offer additional clinical value in the comprehensive management of LDH patients. First, Modic changes may serve as imaging biomarkers that reflect the degree of vertebral endplate degeneration and intervertebral disc pathology, thus aiding in surgical decision-making and risk stratification. Identifying Modic changes preoperatively could help surgeons anticipate technical challenges during discectomy and select the most appropriate surgical approach or extent of decompression (47). Second, Modic changes—especially type I—are often associated with more severe preoperative low back pain and a higher incidence of residual postoperative symptoms. Therefore, evaluating Modic status may help predict patient prognosis beyond herniation recurrence, including pain persistence and recovery of function (48, 49). In such cases, patients may benefit from tailored perioperative management strategies, such as enhanced rehabilitation programs, anti-inflammatory interventions, or adjunctive treatments targeting endplate inflammation. Moreover, the presence of Modic changes may indicate a more advanced degenerative process that could predispose patients to adjacent segment disease or long-term spinal instability (44). Consequently, integrating Modic change assessment into the preoperative evaluation may facilitate long-term treatment planning and improve patient counseling regarding expected outcomes and potential complications.

However, this meta-analysis has some limitations. First, most of the included studies were from China, which may affect the universality of our conclusion. Therefore, additional studies from other countries are needed. Second, all of the included studies were retrospective in design, which may affect the stability of the pooled findings. Third, we were unable to perform more subgroup analyses based on other confounding factors such as age and sex. Finally, only a few studies explored the association between Modic changes and symptom relief after PELD, and we did not define postoperative symptoms as one of our observation indicators.

## Conclusion

Preoperative Modic changes are associated with postoperative recurrence in LDH patients undergoing PELD, and the presence of Modic changes is associated with a significantly higher risk of early and late recurrence. However, well-designed prospective cohort studies or randomized trials are required to validate this association and further clarify any causal mechanisms.

## Data Availability

The original contributions presented in the study are included in the article/Supplementary Material, further inquiries can be directed to the corresponding author.

## Supplementary material

Supplementary File 1

Search strategy in the PubMed database.
