# Supplementary material for: Association between Modic changes and recurrence of lumbar disc herniation after percutaneous endoscopic lumbar discectomy: a meta-analysis
Source: Front Surg. 2025 Nov 27;12:1694557. doi: 10.3389/fsurg.2025.1694557 (PMC12695757; doi:10.3389/fsurg.2025.1694557)

Supplementary figure 1. Subgroup analysis by the recurrence period for the association between the presence of Modic changes and postoperative recurrence in patients with lumbar disc herniation after the percutaneous endoscopic lumbar discectomy.


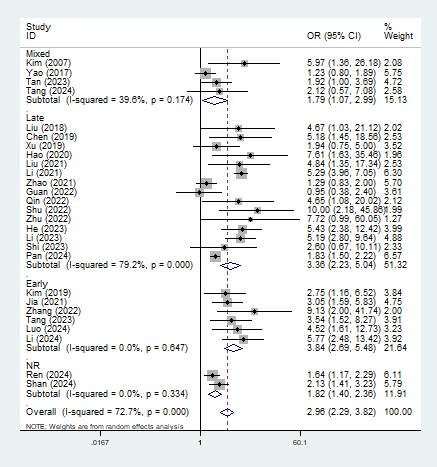


Supplementary figure 2. Subgroup analysis by the recurrence period for the association between the type of Modic changes (II/III vs I) and postoperative recurrence in patients with lumbar disc herniation after the percutaneous endoscopic lumbar discectomy.


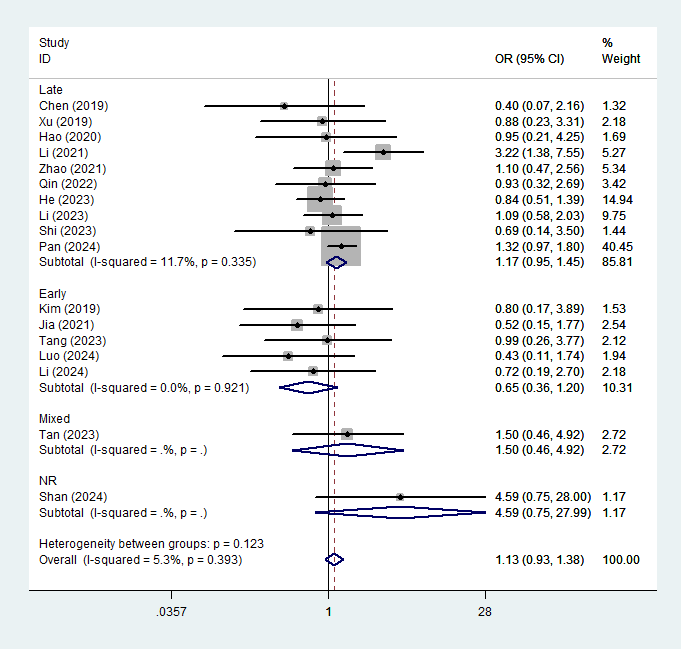

Supplement: Supplementary file 1 [file Datasheet1.docx]
