# Supplementary figures and images for: Association between Modic changes and recurrence of lumbar disc herniation after percutaneous endoscopic lumbar discectomy: a meta-analysis
Source: Front Surg. 2025 Nov 27;12:1694557. doi: 10.3389/fsurg.2025.1694557 (PMC12695757; doi:10.3389/fsurg.2025.1694557)

Search strategy in the PubMed database.


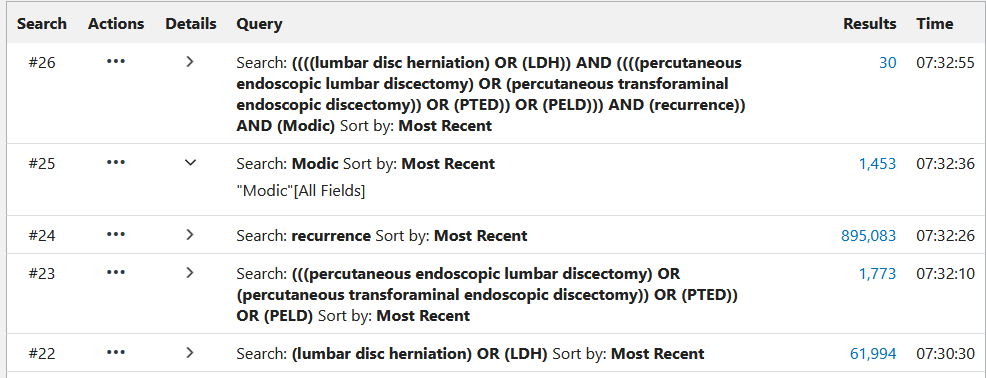

Supplement: Supplementary file 2 [file Datasheet2.docx]
